# Applications of Artificial Intelligence in Drug Repurposing

**DOI:** 10.1002/advs.202411325

**Published:** 2025-03-06

**Authors:** Zhaoman Wan, Xinran Sun, Yi Li, Tianyao Chu, Xueyu Hao, Yang Cao, Peng Zhang

**Affiliations:** ^1^ State Key Laboratory of Common Mechanism Research for Major Diseases Suzhou Institute of Systems Medicine Chinese Academy of Medical Sciences & Peking Union Medical College Suzhou Jiangsu 215123 China; ^2^ Institute of Medicinal Plant Development Chinese Academy of Medical Sciences & Peking Union Medical College Beijing 100193 China; ^3^ Hunan Agriculture University College of Plant Protection Changsha Hunan 410128 China; ^4^ Beijing Key Laboratory for Genetics of Birth Defects Beijing Pediatric Research Institute MOE Key Laboratory of Major Diseases in Children Rare Disease Center Beijing Children's Hospital Capital Medical University National Center for Children's Health Beijing 100045 China; ^5^ College of Life Sciences Sichuan University Chengdu Sichuan 610041 China

**Keywords:** artificial intelligence, drug repurposing, drug‐response prediction, machine learning models, personalized medicine

## Abstract

Drug repurposing identifies new therapeutic uses for the existing drugs originally developed for different indications, aiming at capitalizing on the established safety and efficacy profiles of known drugs. Thus, it is beneficial to bypass of early stages of drug development, and to reduction of the time and cost associated with bringing new therapies to market. Traditional experimental methods are often time‐consuming and expensive, making artificial intelligence (AI) a promising alternative due to its lower cost, computational advantages, and ability to uncover hidden patterns. This review focuses on the availability of AI algorithms in drug development, and their positive and specific roles in revealing repurposing of the existing drugs, especially being integrated with virtual screening. It is shown that the existing AI algorithms excel at analyzing large‐scale datasets, identifying the complicated patterns of drug responses from these datasets, and making predictions for potential drug repurposing. Building on these insights, challenges remain in developing efficient AI algorithms and future research, including integrating drug‐related data across databases for better repurposing, enhancing AI computational efficiency, and advancing personalized medicine.

## Graphical abstract

Artificial intelligence algorithms are transforming drug repurposing by revealing new therapeutic targets and mechanisms. This review highlights the integration of AI frameworks, multimodal datasets, and advanced optimization methods, accelerating the discovery of new indications for existing drugs. These approaches address unmet medical needs and drive advancements in precision medicine by enhancing the efficiency and accuracy of drug repurposing pipelines.

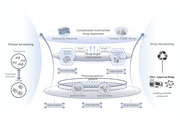

## 1 Introduction

**Figure 1:**
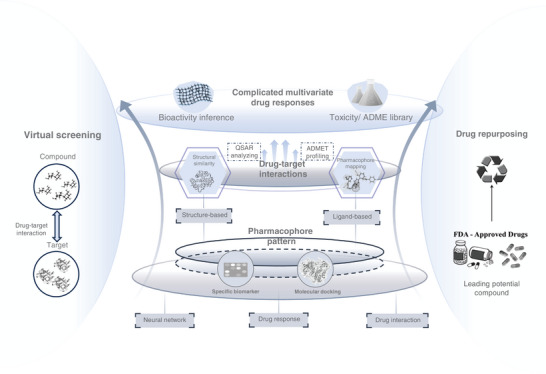
Overview of drug screening processes and mechanisms.

Drug repurposing refers to the strategy of identifying new therapeutic uses for existing drugs that were originally developed for different indications.^[^ ^1^ ^]^ By leveraging the established safety and efficacy profiles of these drugs, this approach can significantly reduce the time, cost, and risk associated with traditional drug development, which provides a valuable pathway for addressing unmet medical needs and expanding the therapeutic options available to healthcare providers.^[^ ^2^ ^]^ Access to drugs is already approved, enabling off‐label clinical studies without the need for new GMP production, lowering trial barriers. As an efficient strategy to address unmet medical needs, several notable examples of successful drug repurposing have been approved by the U.S. Food and Drug Administration (FDA), illustrating the potential of this strategy.^[^ ^3^, ^4^ ^]^ For example, Thalidomide, initially developed and used as a treatment for morning sickness in pregnant women, was withdrawn from the market due to its teratogenic effects.^[^ ^5^ ^]^ However, subsequent research revealed its efficacy in treating multiple myeloma and leprosy, leading to its reintroduction in clinical practice.^[^ ^6^ ^]^ Another prominent example is Sildenafil, which was originally investigated for cardiovascular diseases but was later repurposed and marketed under the brand name Viagra for the treatment of erectile dysfunction.^[^ ^7^ ^]^ Additionally, Minoxidil, originally approved as an antihypertensive agent, was repurposed for the treatment of alopecia due to its hair growth‐promoting effects observed during clinical trials.^[^ ^8^ ^]^ The above clinically validated cases underscore the significant potential of drug repurposing as a strategy to reduce the risks associated with new drug development and can provide patients with new treatment options in a shorter timeframe.^[^ ^9^ ^]^ Consequently, drug repurposing offers a promising approach to identifying potential therapies for conditions that currently lack effective treatments, as well as addressing significant gaps in traditional medical care (**Figure** **1**).

Artificial intelligence (AI) is a fundamental component of computer science, designed to mimic and enhance human cognitive functions, such as learning, reasoning, problem‐solving, decision‐making, and language understanding, and encompasses a wide range of modeling and computational methods, including machine learning, deep learning, natural language processing, as well as heuristic and deterministic optimization algorithms.^[^ ^10^ ^]^ The purpose of AI is to develop specialized computer systems capable of performing complex tasks in a manner similar to human intelligence, such as recognizing patterns and making predictions. With the aid of AI technology, various intelligent products, such as recommendation systems and visualization platforms, have emerged as transformative tools with wide‐ranging applications across numerous industries, including healthcare and pharmaceuticals.^[^ ^11^ ^]^ The implementation of drug repurposing through AI has proven to be notably more cost‐effective and efficient than traditional experimental methods. AI‐driven modeling and computational approaches allow for the rapid evaluation of large compound libraries and the identification of potential drug candidates without extensive laboratory work, thereby significantly reducing the time and financial investment required in the initial stages of drug development. Consequently, this technology greatly enhances and expedites processes that have traditionally been labor‐intensive and time‐consuming.

Leveraging high‐throughput data and multidimensional analytical approaches, AI provides robust technical support for drug repurposing, facilitating advancements in precision medicine and personalized therapies. Through deep learning and molecular modeling techniques, AI can analyze FDA adverse event reports to identify drug side effects that correspond to therapeutic targets for other diseases, thereby uncovering the biological mechanisms underlying these reports.^[^ ^12^ ^]^ For drugs with mechanisms of action that remain incompletely understood, AI‐driven algorithms provide novel insights into potential indications by virtual screening, similarity comparing, or identifying new reports drug response biomarkers.^[^ ^3^, ^13^ ^]^ Furthermore, as drug mechanisms of action often extend beyond their original indications, AI models can facilitate the identification of shared molecular pathways and interconnected networks across diverse therapeutic areas.^[^ ^14^ ^]^ These advancements underscore AI's transformative role in drug repurposing, spanning side effect analysis, mechanism elucidation, and target identification, ultimately accelerating the development of innovative therapeutic strategies.

This review aims to provide a comprehensive and up‐to‐date analysis of the advancements in artificial intelligence for drug repurposing over the past decade. The focus is on how AI facilitates the high‐precision and efficient identification of potential drug candidates by leveraging small molecule compounds and target proteins. Specifically, we introduce key biological and machine learning concepts in several contexts: i) by examining the primary databases and machine learning models commonly employed for drug‐response prediction (DRP); ii) by discussing AI‐driven drug repurposing methods from the perspectives of structural similarity and quantitative structure–activity relationships, while also detailing the inputs, outputs, and usability of the evaluation methodologies utilized; and iii) by exploring drugs with high potential for repurposing and optimization in real‐world data to enhance the therapeutic effectiveness of existing drugs.

## 2 Toolbox of AI Approaches for Drug Repurposing

In this section, we review massive medical databases as essential prerequisites for implementing drug repurposing through AI approaches. Additionally, we outline the main types of data‐driven AI models and algorithms utilized in this context.

### 2.1 Massive Medical Data Resources

Over the world, there are massive databases of medical information available on the Internet, which play a pivotal role in facilitating drug repurposing by mining and analyzing valuable practical implications from these specific datasets, as well as further verifying the derived results (**Table** **1**).

**Table 1:** Database for drug repurposing applications.

Database	Type				URL
	Chemical	Biomolecular	Interaction	Disease	
Drug molecular					
ChEMBL	2431 025 compounds	15 598 targets			https://www.ebi.ac.uk/chembl/
chemDB	5 M small molecules				http://cdb.ics.uci.edu
Pubchem	115 million compounds	304 million biomolecular			https://pubchem.ncbi.nlm.nih.gov/
Drug target					
BindingDB	495 498 small molecules	7032 protein targets	1 142 124 binding		https://www.bindingdb.org/bind/index.jsp
DrugBank	2780 proved drugs	5294 targets	1.3 M drug‐drug interaction		http://www.drugbank.ca
Drug Target Commons (DTC)	4276 compounds	1007 targets			https://osf.io/qdjup
STITCH	30 000 compounds	2.6 million targets	Chemical–protein interaction		http://stitch.embl.de/
Therapeutic Target Database (TTD)	40 000 compounds	3500 targets		500 therapeutic targets	https://idrblab.org/ttd
Drug response					
CCLE	24 anticancer drugs	947 cancer cell line		Cancer	https://sites.broadinstitute.org/ccle/
Comparative Toxicogenomics Database (CTD)	14 923 chemicals	55 359 genes	2 945 493 chemical–gene interaction	3308 diseases	http://ctdbase.org/
L10000	40 000 compounds	978 genes			http://L1000viewer.bio‐complexity.com
PharmGKB	460 proved drugs	247 pathways		5150 clinical annotation	https://www.pharmgkb.org/
GDSC	621 compounds	1000 cancer cell lines	576 758 dose‐response curves	Cancer	http://www.cancerrxgene.org/

Pharmaceutical chemistry is often the most direct reflection of a drug's ability in its targeted therapy. Thus, the relevant data resources in the field of pharmaceutical chemistry help find more perfect drugs for a certain target.^[^ ^15^ ^]^ For example, PubChem (https://pubchem.ncbi.nlm.nih.gov/) is a comprehensive data resource on the biological activities of small molecules, which can provide information on many drugs concerning their chemical structures, properties, biological activities, safety, and toxicity.^[^ ^16^ ^]^ ChEMBL (https://www.ebi.ac.uk/chembl/) provides statistical data on bioactive compounds with drug‐like properties, such as absorption, distribution, metabolism, and excretion (ADME), as well as their toxic properties and their target interactions.^[^ ^17^ ^]^ The current mainstream models of machine learning in the ChEMBL library have demonstrated their outstanding performance in exploring the potential of drugs. DrugBank is also a database that combines detailed drug data with drug target information, including information on drug interactions, mechanisms of action, chemical structures, and pharmacokinetics.^[^ ^18^ ^]^ It has been an essential tool for understanding drug actions and interactions at a molecular level. Embarking on a profound exploration into the intricate realm of drug actions from these data of bioinformatics, such as the databases of genomics, proteomics, and pathway information, is beneficial to unveiling the molecular mechanisms that underlie these pharmaceutical marvels.^[^ ^19^ ^]^ As the stalwart, data‐driven genomic analysis can identify drug targets through omics scrutiny, providing unparalleled insights into the mechanistic effects of drugs.^[^ ^20^ ^]^ The profile data of transcriptomics can navigate the drug potential amidst expression patterns in the dynamic landscape of drug discovery, often being a powerful tool for gauging and delving into the potentials of pharmaceutical interventions. Online Mendelian Inheritance in Man (OMIM) is an extensive catalog of human genes and genetic disorders, including information on the phenotypic effects of mutations and the genetic basis of diseases. Hence, OMIM is often employed to identify potential targets of a drug by linking genetic variations to diseases.^[^ ^21^ ^]^

Apart from the above‐mentioned databases for a broad spectrum of purposes, there also exist specific ones. For example, Protein Data Bank (PDB, https://www.rcsb.org/) is a comprehensive database of 3D structural data of biological molecules, such as proteins and nucleic acids, hence is often used to reveal the molecular mechanisms of drug‐target interactions.^[^ ^22^ ^]^ Particularly, the PDB derived databases such as PDBbind and BioLiP2 are curated to provide detailed information of protein–ligand binding affinities, binding modes, as well as functional annotation.^[^ ^23^ ^]^ The Library of Integrated Network‐based Cellular Signatures (LINCS) L1000 dataset is a valuable resource for drug repurposing studies,^[^ ^24^ ^]^ including the transcriptional responses of human cells to various drug perturbations at a genomic scale. Since L1000 captures the gene expression profiles for a large number of drugs, it is often employed to identify new therapeutic uses for existing drugs.^[^ ^25^ ^]^ It is certain that different types of data intricately shape the interpretability of drug repurposing algorithms, revealing a range of strategies for discovering new therapeutic applications for existing drugs.^[^ ^26^ ^]^ However, it is still a challenge how to integrate and fuse the drug‐related information hidden in all the databases so that they are better utilized to discover more new uses for the existing drugs, as well as accelerate drug development and improve patient care.

Indeed, variations in data formats, measurement techniques, and reporting standards can lead to inconsistencies that complicate the process of combining datasets. To address these challenges, researchers need to develop robust data integration and standardization protocols, involving harmonizing data from various sources to ensure compatibility and comparability, as well as employing advanced data preprocessing techniques to handle missing or inconsistent information. Argelaguet et al. developed a multiomics factor analysis (MOFA) that can distinguish between the heterogeneity axes common to multiple models and the heterogeneity axes specific to a single data model to identify the main sources of variation in multiomics data sets and infer a set of (hidden) factors.^[^ ^27^ ^]^ Liu et al. constructed a hybrid multiview and multiscale graph dual attention network (HMM‐GDAN) for cancer drug response prediction based on multiview graph learning of gene profiles to capture the complementary information of views and jointly emphasize the importance of each view to form high‐level representations, and verified the effectiveness of the multiview and multiscale strategy on the GDSC2 dataset.^[^ ^28^ ^]^ Chen et al. compiled a comprehensive multimodal dataset of baseline antimalarial activity, and evaluated the predictive performance of two fingerprint‐based machine learning models (RF::Morgan and XGBoost::Morgan), four graph‐based deep learning models (GCN, GAT, MPNN, and Careful FP), and three corepresented deep learning models (FP‐GNN, HiGNN, and FG‐BERT). The MalariaFlo server has been developed to effectively predict novel phase III antimalarial drugs, which have been validated through experimental testing.^[^ ^29^ ^]^ Currently, number of web‐based platforms are publicly available for deploying models to perform drug repurposing.^[^ ^30^ ^]^ These platforms integrate computational tools, databases, and machine learning algorithms to streamline repurposing analysis. In addition to the previously mentioned DrugBank and LINCS, the Broad Institute provides the CLUE (Connectivity Map) platform, which utilizes transcriptomic data to identify potential drug repurposing opportunities by comparing disease gene expression profiles with drug‐induced gene expression profiles.^[^ ^31^ ^]^ Furthermore, analytical tools such as GNINA and DeepChem can be hosted or integrated into web‐based environments to perform tasks like virtual screening, thus further supporting drug repurposing research.^[^ ^32^ ^]^

### 2.2 The Types of AI Models and Algorithms in Drug Repurposing

**Figure 2:**
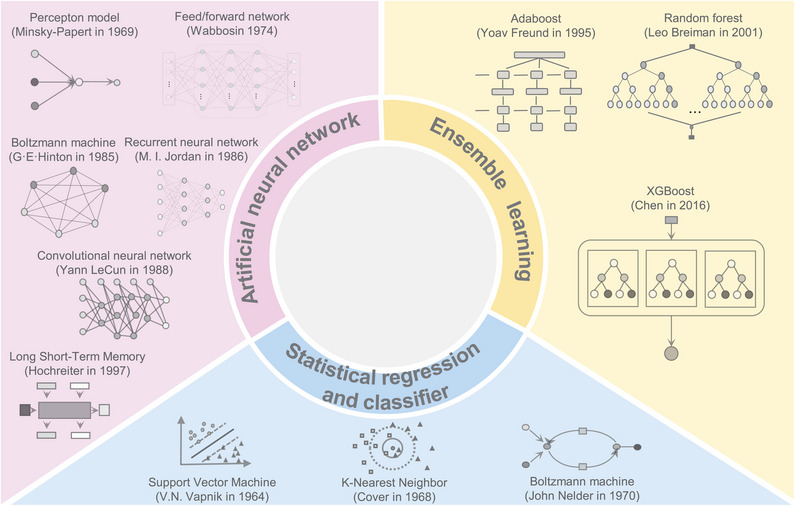
Comprehensive overview of artificial intelligence in machine learning.

Since a majority of the drug‐related datasets contain large‐volume data with different modes and complex heterogeneity, it is difficult to apply the traditional statistical analysis methods to accurately predict drug actions.^[^ ^33^ ^]^ In contrast, AI algorithms excel in handling and analyzing large datasets, especially for drug repurposing based on the vast amounts of biomedical databases available. On the one hand, some AI algorithms can be applied to sift through the existing theoretical research, the clinical trial results, the patient records, and the genetic information, which can be combined with the existing databases to provide richer verification for the subsequent data analysis. On the other hand, AI algorithms have significantly advanced the fields of drug discovery by making predictions based on appropriate optimization models and identifying inherent patterns in terms of the physical and chemical properties of drugs and their active mechanisms. From this perspective, it is plausible that AI models and algorithms can autonomously carry out the complex tasks involved in drug repurposing (**Figure** **2**).

#### 2.2.1 Algorithms for Linking Drug Compounds with Potential Targets

As one of the most popular AI methods in drug repurposing, similarity‐based approaches often employ supervised learning models and algorithms, such as linear regression, logistic regression, support vector machines (SVM), and Boltzmann networks, to process and analyze labeled data, which are trained to identify patterns of drug properties and annotation associations.^[^ ^34^ ^]^ Such supervised algorithms can construct drug‐target association patterns and predict potential novel target associations. In contrast, unsupervised learning models, such as K‐means clustering and principal component analysis (PCA), are typically used to extract representative attributes and hidden patterns from unlabeled drug features, enabling the inference of potential targets based on drug similarity.^[^ ^35^ ^]^ Generative Adversarial Networks (GANs) are a class of machine learning frameworks designed to generate new data samples that are similar to a given dataset, consisting of two neural networks: a generator and a discriminator.^[^ ^36^ ^]^ Through an adversarial training process, the generator learns to produce increasingly realistic data, and the discriminator becomes better at distinguishing real from synthetic data, showing immense potential in drug repurposing due to their ability to generate novel molecular structures and predict drug interactions. By generating synthetic interaction data, the flexibility and scalability of these deep learning models can be applied to predict potential interactions between the drugs and the biological targets, to optimize the pharmacokinetic properties of the existing drugs, or to modify the drug structures with improved profiles.^[^ ^37^ ^]^

#### 2.2.2 Algorithms for Revealing Indications Linked Known Mechanism

Among artificial intelligence (AI) methods, artificial neural networks (ANNs) are prominently utilized in drug repurposing to decode complex drug systems and uncover novel therapeutic applications of existing drugs based on prior knowledge and data.^[^ ^38^ ^]^ Recurrent Neural Networks (RNNs), with their ability to capture sequential dependencies in dynamic datasets such as time‐series gene expression, drug response trajectories, or signaling pathways, play a critical role in modeling temporal drug modulation processes.^[^ ^39^ ^]^ The feedback loops allow the retention of information across time steps, facilitating the prediction of downstream biological effects caused by upstream perturbations, which enables RNNs to identify dynamic therapeutic responses and uncover potential benefits over time. Convolutional Neural Networks (CNNs), renowned for their spatial feature extraction capabilities, are extensively applied in domains, such as protein structure prediction, molecular docking, and drug‐target interaction analysis. By identifying hierarchical patterns and spatial relationships, CNNs help elucidate the structural compatibility between drug molecules and biological targets, thereby advancing the discovery of novel mechanisms of action and optimizing drug repurposing pipelines. Multilayer Perceptrons (MLPs), as feedforward networks adept at modeling nonlinear input–output relationships, are widely used for tasks like drug classification and regression.^[^ ^40^ ^]^ By analyzing high‐dimensional drug data, including chemical properties, pharmacokinetics, and genomic profiles, MLPs can reveal latent patterns linked to new therapeutic effects, offering valuable insights into alternative drug indications. Different neural network approaches collectively enhance drug repurposing efforts by decoding sequential dynamics, spatial features, and intricate relationships within biological systems, which enable the decoding of intricate biological mechanisms and the identification of novel pathways for drug efficacy. Integrating these methods into drug repurposing pipelines not only accelerates the discovery process but also enhances the precision of developing treatments for unmet medical needs.

#### 2.2.3 Algorithms for Identifying Clinical Effects in Real‐World Evidence

With the proliferation of drug repurposing research and experimental validations, Natural Language Processing (NLP) has emerged as a pivotal AI technology for extracting and structuring knowledge from unstructured data sources. NLP models enable systematic mining of vast biomedical resources, such as scientific publications, clinical case reports, and electronic health records, to uncover previously unrecognized therapeutic applications. Transformer‐based architectures like BERT and GPT, alongside Word Vector models like Word2Vec, excel in capturing semantic nuances and contextual relationships. These capabilities facilitate connections between molecular data, biological pathways, and clinical outcomes, offering fresh insights into drug mechanisms and indications. For instance, BERT and GPT can analyze large‐scale textual corpora to identify drug‐related side effects and novel indications, while Word2Vec aids in modeling molecular similarity to infer shared therapeutic properties among compounds.^[^ ^41^ ^]^ In addition to NLP approaches, heuristic and evolutionary optimization algorithms, such as genetic algorithms, simulated annealing, and particle swarm optimization, have been employed to address the complex optimization challenges inherent in drug repurposing. These algorithms, free from stringent assumptions about objective functions and constraints, are particularly adept at tackling nonlinear, high‐dimensional optimization problems.^[^ ^42^ ^]^ Furthermore, regularized optimization models integrated with deterministic algorithms, particularly for nonnegative matrix factorization, have demonstrated superior efficiency in clustering and dimensionality reduction tasks. These unsupervised learning methodologies hold promise for optimizing drug repurposing workflows by balancing computational complexity and prediction accuracy.^[^ ^43^ ^]^ By synergizing NLP's capability to derive actionable insights from unstructured data with advanced optimization algorithms for model refinement, AI‐driven frameworks are transforming the landscape of drug repurposing, not only accelerating the discovery pipeline but also enhancing the precision of therapeutic predictions, ultimately addressing unmet clinical needs and supporting the advancement of personalized medicine.^[^ ^44^ ^]^

## 3 Application Scenarios of AI Algorithms for Drug Repurposing

**Figure 3:**
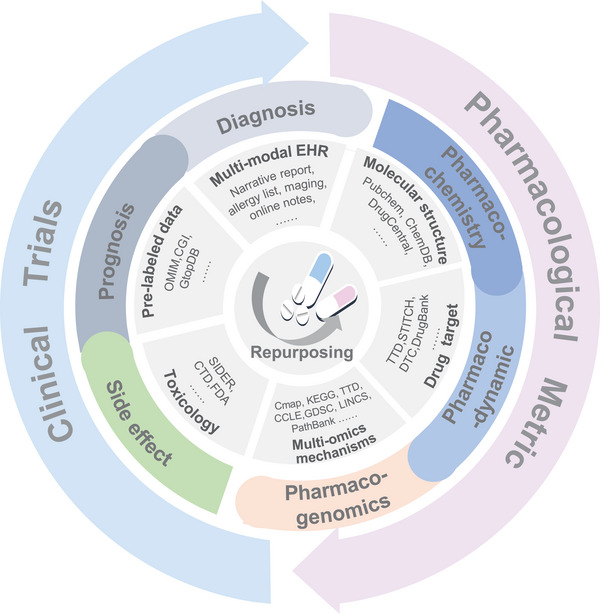
The direction of artificial intelligence learning mechanism and clinical optimization of drug repurposing.

In this section, we outline strategic approaches leveraging advanced AI technologies to purpose drugs and expand their therapeutic applications. By integrating real‐world evidence, the power of AI models and algorithms enhance the therapeutic potential of existing drugs and establish a comprehensive framework for identifying new indications (**Figure** **3**).

### 3.1 Transforming Side Effects into Therapeutic Opportunities

Drug side effects are often viewed as unintended outcomes of treatment, but can also reflect a broader interaction between the drug and biological systems. For example, side effects such as weight loss, skin reactions, or blood pressure changes may indicate a drug's potential in treating unrelated conditions.^[^ ^45^ ^]^ Pharmacogenomics focuses on understanding the genetic basis of variation in drug responses between individuals. By combining pharmacological data with genomic information, researchers can assess how individual genetic variations influence the efficacy and safety of drugs.^[^ ^46^ ^]^ Currently, the focus has been on constructing multiomics drug response data resources for various clinical scenarios, which has propelled the development of AI algorithms designed to tailor drug treatments based on an individual's genetic profile.^[^ ^47^ ^]^ Pacini et al. annotated 930 cancer cell lines with multiomics data, and by combining clinically meaningful dependency marker associations with protein–protein interaction networks, they constructed an anticancer target priority map, which can be applied to identify 370 anticancer priority targets for 27 types of cancer.^[^ ^48^ ^]^ Sakellaropoulos et al. trained a deep neural network on 1001 cancer cell lines to predict the drug responses and validated the performance superiority of the developed AI algorithm in precise tumor therapy on multiple clinical groups.^[^ ^49^ ^]^ Interpretable deep learning translation GWAS and multiomics discoveries have enabled pathobiological identification and drug reuse in Alzheimer's disease, cardiovascular and cerebrovascular diseases, and multiple cancers.^[^ ^50^ ^]^ In the field of tumor drug repurposing, AI models based on a large number of small molecule databases promote the discovery of anticancer uses of nontumor drugs. For instance, Corsello et al. created a molecular barcoding technology, which has become a public resource for drug screening using PRISM (https://depmap.org/repurposing), containing 4518 drug growth inhibition activities in 578 human cancer cell lines.^[^ ^51^ ^]^

Leveraging disease databases and biochemical interaction data, researchers can cross‐reference pharmacological properties to uncover therapeutic pathways and targets linked to drug side effects. Quantitative Structure‐Activity Relationship (QSAR) modeling further enhances this process by correlating chemical structures with biological activities, facilitating the identification of new therapeutic applications for existing drugs.^[^ ^52^ ^]^ By taking the overall structural properties of the molecule as a parameter (without considering the 3D structure of the molecule), annotated regression analysis is performed on the physiological activities (activity, toxicity, pharmacokinetic properties, etc.) of the molecule, and the correlation between its chemical structure and physiological activity is quantified. In terms of their different uses, these types of modeling methods can be divided into QSAR, QSTR (toxicity), QSPR (pharmacokinetics), etc.^[^ ^53^ ^]^ An important advantage of the deep QSAR methods over the traditional QSAR methods is that they may more effectively perform multiobjective optimization tasks and improve prediction accuracy by using knowledge transfer, such as the concurrent use of different data available for different tasks.^[^ ^54^ ^]^ Because it does not consider the 3D conformation of molecules, 2D‐QSAR usually does not require complex molecular structure optimization and superposition and has high computational efficiency. Therefore, QSAR drug inference based on deep learning can often achieve an accurate evaluation of drug efficacy in a limited computational space. These models can automatically learn the relevant features from the raw molecular structures, such as the molecular graphs and the SMILES strings. Thus, they can capture the intricate structural details.^[^ ^55^ ^]^ Hu et al. generated a fixed‐size underlying feature from an end‐to‐end encoder‐decoder model and connected it with a convolutional neural network (CNN) architecture to train a robust and stable model for compound activity prediction.^[^ ^56^ ^]^ Zhang et al. constructed ReLeaSE (Reinforcement Learning for Structural Evolution) and integrated two deep neural networks based on deep learning and reinforcement learning (RL) methods, where generation and prediction were performed only by using the strings in Simplified Molecular Input Line Entry System (SMILES). Therefore, QSAR model, particularly combined with natural language processing (NLP), machine learning, and other network pharmacology, can analyze vast amounts of clinical data to explore the underlying mechanisms of side effects identified during development, uncovering unrecognized therapeutic potentials.

Repurposing drugs based on their side effects plays a significant role in addressing real‐world clinical challenges and unmet medical needs, not only highlighting the multifunctionality of existing drugs but also underscoring the importance of re‐evaluating the unintended effects of treatments as a source of therapeutic innovation. One of the most well‐known examples of a drug repurposed due to its side effects is aspirin, originally developed as an analgesic and anti‐inflammatory agen, which observed blood‐thinning effect led to its widespread use in preventing cardiovascular events.^[^ ^57^ ^]^ Currently, AI algorithms are being employed to optimize aspirin dosage by analyzing large‐scale clinical data and identifying the subgroups that benefit most from its cardiovascular protective effects.^[^ ^58^ ^]^ Furthermore, timely identification and discontinuation of drugs is the cornerstone of clinical management. For example, with the help of disease‐related adverse event (AE) reports from the US Food and Drug Administration (FDA) Adverse Event Reporting System (FAERS) database, the risk characteristics of drugs can be obtained by analyzing the comprehensive signal distribution of ADR signal detection results by disproportionation analysis, which provides a comprehensive overview of the current AP culprit‐drug from the perspective of pharmacovigilance. Thus, it can provide reference information for clinical practice.^[^ ^59^ ^]^ Letswaart et al. used in vitro secondary pharmacology of common targets of 2134 marketed drugs from FDA adverse event reports to establish a random forest model to predict the occurrence of adverse reactions from an in vitro pharmacological perspective.^[^ ^60^ ^]^ In addition, even for the marketed drugs, there are still risk factors for adverse reactions in the real world, such as patients who are still at risk of newly acquired human immunodeficiency virus type 1 (HIV‐1) resistance despite the introduction of combination antiretroviral therapy (cART).^[^ ^61^ ^]^ By identifying drug side effects and applying heuristic AI algorithms to uncover the underlying biological interactions, new therapeutic strategies can be discovered. This systematic analysis forms the basis for innovative drug development and creates new opportunities for clinical applications.

### 3.2 Exploring New Indications Based on the Original Mechanism

By investigating the known biological pathways and targets of a drug, it is possible to uncover other conditions where its original mechanism of action may be beneficial. Through drug‐target interaction screening based on cellular drug responses, new indications can be identified, enabling the repurposing of existing drugs to address a broader range of therapeutic needs and offer new opportunities for clinical application.

High‐throughput screening allows for the rapid assessment of thousands of compounds against a wide range of biological targets enabling the identification of new interactions between the existing drugs and the previously unexplored targets.^[^ ^62^ ^]^ By systematically analyzing the extensive datasets, the high‐throughput screening may identify new uses for the existing drugs based on their molecular interactions and effects on cellular processes.^[^ ^63^ ^]^ Amadori et al. presented a path of drug repurposing to identify immunotherapies for atherosclerotic cardiovascular disease (ASCVD) by comparing the inflammatory signatures to large‐scale gene expression data of the LINCS L1000 dataset.^[^ ^64^ ^]^ Subramanian et al. analyzed the connectivity scores between the gene expression profiles of diseases and the drug‐induced profiles.^[^ ^65^ ^]^ Lamb et al. applied machine learning techniques to analyze connectivity scores and predict the potential drug‐disease pairs and successfully predicted new indications for several FDA‐approved drugs from the utility of transcriptional data.^[^ ^66^ ^]^ Jia et al. developed a deep variational autoencoder (VAE) model to compress thousands of genes into latent vectors in a low‐dimensional space. Then, these encoded vectors were used to accurately impute drug responses, which outperformed the standard signature‐gene‐based approaches, apart from appropriate control of overfitting.^[^ ^67^ ^]^ Considering the nuanced facts that a single drug often corresponds to multiple targets and downstream pathways, the prediction of potential drug targets not only aids in understanding drug actions but also serves as a compass when identifying the drugs tailored for diseases with well‐defined molecular mechanisms.^[^ ^68^, ^69^ ^]^

Continued advancements in the integration of datasets, computational modeling, and collaborative research further enhanced the field of drug repurposing, ultimately improving patient outcomes and accelerating the development of new treatments.^[^ ^70^ ^]^ Yang et al. built an MD‐FIS deep learning model based on a limited experimental data set and trained the model on the data of oral rapid disintegrating membrane (OFDF) and the oral sustained release matrix tablets (SRMT). Compared with MLR, PLSR, SVM, ANNs, RF, and k‐NN, the deep learning framework can better identify the complex correlation between drug formulation and in vitro properties. Based on pharmacokinetics and pharmacodynamics, Marcel et al. trained two random forest‐based ensemble models to predict the ATC classes by structural and physiochemical properties.^[^ ^71^ ^]^ There is growing evidence that psychotropic drugs have anticancer potential based on the extrapolation of their drug properties.^[^ ^72^ ^]^ Detailed information about the pharmacokinetics, pharmacodynamics, and adverse effects of these drugs is readily accessible, allowing researchers to make informed decisions regarding their potential new uses.^[^ ^63^ ^]^ El‐Hachem proposed an unbiased, integrated computational pharmacogenomics approach, which integrated the drug structure information, high‐throughput drug perturbations, and a framework of drug sensitivity profiles. Thus, it can be applied to identify the related and novel drug–drug relationships to infer scalable drug classifications that rely only on the essential drug characteristics.^[^ ^73^ ^]^ Despite this method being suitable for studying experimental drugs with potential new targets, challenges arise from the inherent data heterogeneity across different sources, introducing complexities during the integration and standardization of large‐scale data analyses.

By exploring the alternative targets and underlying mechanisms, researchers can also find the commonalities between different diseases or conditions, which is beneficial to uncover new therapeutic possibilities and guide the selection of potential repurposing candidates when the mechanisms of action are congruent with the pathophysiology of the targeted diseases.^[^ ^74^ ^]^ This result not only aids in identifying suitable drugs for repurposing but also in optimizing their uses across various disease contexts.^[^ ^75^ ^]^ DrugCell, an interpretable deep‐learning model of human cancer cells, was trained on the responses of 1235 tumor cell lines to 684 drugs, and was applied to predict responses to the therapy. By learning the biological mechanisms underlying these drug responses, the proposed model can provide a blueprint for constructing interpretable models for clinical practice.^[^ ^76^ ^]^ Using the well‐trained models, the signatures associated with the imputed drug response can also be further investigated for the actual treatment outcome, including cell line origins, somatic mutations and tumor mutation burdens, tumor microenvironment, and confounding factors. Using the Functional Representation of Gene Signatures (FRoGS), one can perform comparative analyses on omics datasets to train a deep learning model. It was demonstrated that novel relationships between the obtained gene signatures can be revealed from the large‐scale omics studies of compounds, cell types, disease models, and patient cohorts when using the predictive networks pre‐equipped with knowledge of gene function.^[^ ^77^ ^]^ Within a deep transfer learning framework, scDEAL can predict cancer‐drug responses at the single‐cell level by integrating the large‐scale bulk cell‐line data and harmonizing the drug‐related bulk RNA‐seq data with the scRNA‐seq data.^[^ ^78^ ^]^ Through these strategies, the therapeutic utility of a drug can be greatly enhanced, offering promising avenues for both drug development and clinical application.

### 3.3 Identifying Novel Biomolecular Targets for Existing Drugs

Discovering novel biomolecular targets for existing drugs is a key strategy in drug repurposing, offering the potential to expand therapeutic applications and address unmet medical needs. Drug networks based on chemical structure similarity can be employed to identify drugs with similar mechanisms of action or potential off‐target effects. Therefore, identifying the clusters of compounds with similar biological activities to pharmacological characteristics can offer unique insights into drug similarity.^[^ ^79^ ^]^ Since molecular docking can assess the binding modes and affinities between a drug and its target protein, some scoring functions, such as AutoDock or Glide, are used to calculate the binding energy between a drug and target and predict the likelihood of interaction.^[^ ^80^ ^]^ Furthermore, combined with kernel density estimation, a probability density model is constructed to obtain the properties associated with pharmacological targets based on the drug molecular fingerprint as a 3D similarity measure (Q‐Q matrix and A‐L vector) of molecular representation, which can be used to measure the quasidistance between ligands.^[^ ^81^ ^]^ SwissTargetPrediction (www.swisstargetprediction.ch) is such a network tool of anticipating the potential drug targets.^[^ ^82^ ^]^ Graph‐based machine learning algorithms are widely used to identify more than 2D drug and target structures.^[^ ^83^ ^]^ Wong et al. developed an interpretable substructure‐based graph neural network model to determine the antibiotic activity and human cytotoxicity profiles of 39 312 compounds and predicted the antibiotic activity and cytotoxicity of 12 076 365 compounds with higher accuracy.^[^ ^40^ ^]^ They also demonstrated that chemical substructures associated with antibiotic activity can be identified and employed to predict the structural categories of antibiotics. CycleGAN, a generation model of molecular substructure trees, can efficiently extract novel molecular and protein structural features to effectively improve molecular performance.^[^ ^84^ ^]^ Azagury et al. developed a decision tree model by literature text mining, which can capture the drug pairs with biological synergies and synergistic chemical self‐assembly, and generate a database with 1985 drug pairs involving 70 cancers.^[^ ^85^ ^]^ PIGNet is a physics‐informing algorithm by augments a broader range of binding poses and ligands to the training data, to provide insights for further ligand optimization. It was demonstrated that it outperformed docking and screening powers the previous methods in the comparative assessment of scoring functions (CASF) 2016.^[^ ^86^ ^]^

**Figure 4:**
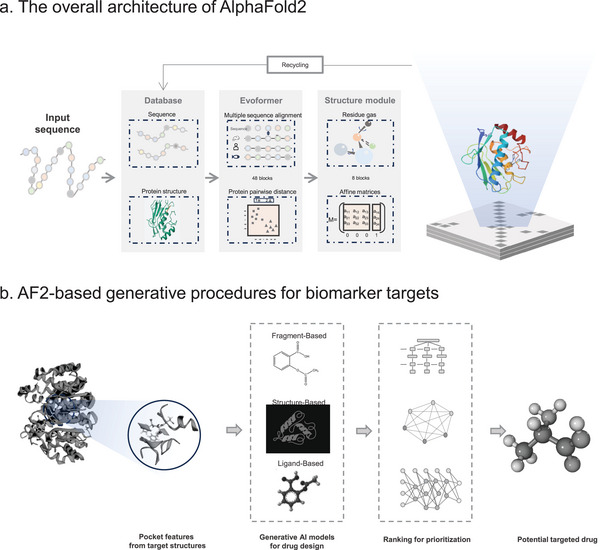
Comprehensive overview of artificial intelligence in machine learning. a) The overall architecture of AlphaFold2. b) AF2‐based generative procedures for biomarker targets.

As a new standard in CASP (Critical Assessment of protein Structure Prediction) competitions, AlphaFold series (AF), developed by DeepMind, has made groundbreaking contributions by achieving near‐experimental accuracy in predicting protein structures.^[^ ^87^ ^]^ Particularly, it utilizes deep learning to predict the distances between amino acids and the angles of chemical bonds in proteins, to produce 3D models with higher accuracy (**Figure** **4**). By applying the state‐of‐the‐art AlphaFold2, it is possible to predict almost the entire human proteome (98.5% of human proteins), with 58% of the residues having high confidence. On this basis, interpretable indicators were developed, facilitating stronger multidomain predictions and identifying potentially disordered regions.^[^ ^88^ ^]^ The combination of AF and AI promotes the inherent flexibility of peptide‐based drugs and heterogeneous structural prediction of conformational preferences between free and bound states, providing new opportunities for highly specific, potent, and selective peptide‐based drug discovery.^[^ ^89^ ^]^ Additionally, molecular docking models based on the chemical properties of drugs can also predict how small molecules (ligands) bind to their target proteins by similarity‐based methods of identifying potential therapeutic properties. Recently, the proposed ligand–protein cofolding algorithm can not only simulate ligand–protein binding but also the conformational changes that occur between proteins and ligands during binding, which is crucial for understanding drug‐receptor interactions.^[^ ^90^ ^]^ Rube et al. adopted interpretable machine learning models to predict protein‐ligand binding affinity by integrating large‐scale sequencing information. This approach enabled the identification of key features influencing binding affinity and provided a deeper understanding of the mechanisms underlying protein–ligand interactions.^[^ ^91^ ^]^ AlphaFill leverages ligands and cofactors to refine the protein models predicted by the AlphaFold protein structure database, addressing the lack of constraints on the coordinates of small molecules that are crucial for molecular structure or function.^[^ ^92^ ^]^

AI algorithms, particularly those leveraging machine learning, natural language processing (NLP), and network pharmacology, are capable of analyzing large‐scale biological and pharmacological datasets to predict new targets for existing drugs.^[^ ^93^ ^]^ DeepION, a deep learning method specifically designed for ion image representation, was applied to the identification of colocalization ions and isotopic ions, and the validity of this ion image representation was verified by the mass spectrometry imaging (MSI) experiments in rat brain tissues. Thus, it can serve as an important tool for discovering biomarkers and developing new drugs.^[^ ^94^ ^]^ By mining the chemical structure data from PubChem, Keiser et al. applied the chemical similarity methods to predict off‐target effects of drugs, and found unexpected drug‐target interactions that suggest new uses for several FDA‐approved drugs.^[^ ^74^ ^]^ The large‐scale MD‐based datasets PLAS‐20k with 97 500 independent simulations on a total of 19 500 different protein–ligand complexes showed a strong correlation with the available experimental values, performing better than the docking scores.^[^ ^95^ ^]^ Using computer perturbation to measure drug–drug similarity in multimodal datasets, the MOVE model identified pharmacologic links for 20 common drugs in type 2 diabetes and uncovered new connections between metformin and the gut microbiome, as well as contrasting molecular effects of simvastatin and atorvastatin.^[^ ^96^ ^]^ Within the machine learning framework, DRIAD (Drug Repurposing In AD) quantified the potential associations between the pathology of AD severity (the Braak stage) and applied these molecular mechanisms to produce a ranked list of possible repurposing candidates for the 80 FDA‐approved and clinically tested drugs.^[^ ^97^ ^]^ Therefore, AI can predict how drugs modulate biological networks and identify unexpected targets that could offer new therapeutic opportunities, enabling the exploration of both off‐target effects and secondary targets, which may contribute to a drug's therapeutic efficacy in different disease contexts.

### 3.4 Integrating AI and Real‐World Evidence to Optimize Drug Repurposing

Advanced AI models and algorithms have leveraged correlations between chemical structures, pharmacological activities, and reported adverse effects to predict the likelihood of specific reactions in different patient populations. Therefore, it is sure that they have a strong ability to identify compounds with potentially fewer side effects through early risk identification and ongoing monitoring.^[^ ^98^ ^]^ Further combination of computational models and rich drug databases like DrugBank drives greater innovation in the field of drug repurposing, such as the strategies of more efficient and cost‐effective drug development. By integrating DrugBank data with other biological and clinical datasets, researchers have identified new therapeutic uses for the existing drugs, experimentally validated the predicted results by the AI models, and proposed new strategies for mitigating adverse drug reactions.^[^ ^99^ ^]^

#### 3.4.1 Interventional Pharmacology for Clinical Prognosis

**Figure 5:**
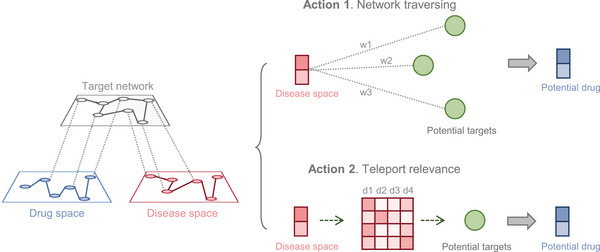
Cross‐referencing of biomedical databases and pharmacological properties contributes to the drug repurposing.

Interventional pharmacology is one of medicine's most powerful weapons in the fight against disease. Bioactivity‐based drug metrics tend to use bioassay data to screen compounds that show desired biological activity in relevant assays based on the biological activity observed in different biological settings (**Figure** **5**). Thus, this technique can facilitate the identification of new therapeutic uses. Chou et al. combined an AI‐based QSAR model with a PBPK model to build an AI‐assisted physiologically based pharmacokinetic (PBPK) model that simulates the tumor‐targeted delivery efficiency (DE) and biological distribution of NPs.^[^ ^100^ ^]^ The predicted results of the AI PBPK model have been shown to have a good correlation with the pharmacokinetic characteristics of different NPs measured in the tumor after intravenous injection (R2≥0.70 in 133 of 288 groups of data), hence providing an efficient drug screening tool that can quickly predict the delivery efficiency of NP based on its physical and chemical properties, without relying on animal training data sets. Due to the potentially devastating side effects of drugs, pharmacovigilance has become an important scientific issue in the monitoring, detection, and prevention of adverse drug reactions (ADR). The combination of the pharmacological properties with the clinical and toxicological databases plays a crucial role in predicting adverse drug reactions (ADRs) and assessing drug safety, as well as extending to postmarketing surveillance of ADR in real‐world populations. Lounkine et al.^[^ ^101^ ^]^ used PubChem to predict adverse drug reactions (ADR) and identified the therapeutic potential of several drugs with previously unrecognized controllable side effects. Letinier developed an automated system based on supervised learning to code unstructured text of patient adverse reaction reports.^[^ ^102^ ^]^ The drug‐likeness properties of lead compounds were predicted by ADME analysis based on molecular docking and binding free energy studies.^[^ ^103^ ^]^

#### 3.4.2 Drug Conversion Based on Electronic Medical Records

Large‐scale longitudinal case‐control studies and systematic medical record reviews based on the AI models combined with electronic medical records and drug knowledge maps have been integrated to identify the risk factors associated with adverse reactions to drugs, which is beneficial to improve patient care and prevent drug resistance and treatment failure. With resource‐rich environment support in terms of efficient identification and validation of repurposing candidates, researchers have made significant progress in repurposing the existing drugs.^[^ ^104^ ^]^ Clinical data, encompassing electronic health records, patient outcomes, adverse event reports, and various diagnostic, treatment, and investigation records, all constitute a valuable repository, offering a direct connection to the real‐world efficacy and safety profiles of drugs.^[^ ^105^ ^]^ The AI models and algorithms can be further applied to analyze the real‐world evidence from electronic health records (EHRs), patient registries, and other sources to uncover more new indications for the existing drugs, especially by examining how the drugs are used in real‐world settings and their outcomes. The accumulation of more available data on drugs further provides an opportunity to use AI models and algorithms to improve the safety of the drugs. Using a combination of adverse event reports, EHR, and laboratory experiments, 1.8 million adverse event reports were mined to obtain insights, which demonstrated a QT interval‐prolonging drug–drug interactions (QT‐DDI).^[^ ^106^ ^]^ The transparency in the safety and efficacy profiles of the marketed drugs provides a reliable foundation for the repurposing efforts of drugs.^[^ ^107^ ^]^ Prof. L. B. Sheiner, the pioneer of modern pharmacometrics, envisioned the drug development process as a series of learn‐confirm cycles with model‐based analyses and simulations at the core of the learning process and led to the concept of model‐informed drug development (MIDD) that is being evaluated and promoted by regulatory agencies, pharmaceutical industry, academic scientists, and clinicians.^[^ ^108^ ^]^ A network meta‐analysis of schizophrenia combining randomized controlled trials and real‐world data has been conducted using virtual screening, which can successfully estimate the efficacy and effectiveness of antipsychotic drugs in preventing schizophrenia relapse.^[^ ^109^ ^]^ Clearly, with the use of clinical cohorts and real‐world data, these results from the AI models and algorithms can advance a deeper understanding of the generality of RCT results in clinical practice, and inform valuable guidelines for priority prescribing.

## 4 Challenges and Future Directions

In this section, on the basis of reviewing the existing results, we focus on addressing the challenges in drug repurposing and the research directions in the future, apart from those stated in the previous sections.

### 4.1 The Opportunities and Challenges of Computing Resources

The practical application of AI in drug repurposing is significantly enhanced by robust data integration and the development of advanced machine learning models. Integrating diverse datasets is crucial for providing a comprehensive view that supports accurate predictions, while sophisticated AI algorithms can manage the complexity and volume of data to uncover new therapeutic insights. However, despite the availability of vast datasets, computational analysis of millions of molecules based on known drugs still faces significant limitations in identifying new therapeutic applications. The success of drug repurposing algorithms heavily depends on the quality and completeness of the underlying data. Incomplete data—characterized by missing or inadequate information—can arise from limited experimental data, gaps in clinical records, or omissions in biological databases. Such data incompleteness can lead to models that fail to accurately represent biological processes, resulting in poor predictive performance. Moreover, nonrandom data absence can introduce bias into these models. For example, if certain populations are underrepresented in clinical trial data due to specific interventions, the generalizability and applicability of the model across diverse populations can be severely compromised. Another significant challenge in the field of drug repurposing is the presence of imbalanced samples, where different categories within a dataset are disproportionately represented. Algorithms trained on imbalanced data are prone to favor the majority class, which can lead to high false‐negative rates. This means that potential drug repurposing opportunities may be overlooked, and the model may fail to detect rare but crucial patterns. For instance, a model that is trained predominantly on data from widely studied diseases may underperform when predicting drug repurposing opportunities for less common conditions. The heterogeneity and challenges presented by large‐scale drug databases pose substantial obstacles to the effective application of drug repurposing algorithms. Incomplete data can result in biased and inaccurate models, while imbalanced samples can skew predictions and diminish the model's sensitivity to rare but important patterns. Addressing these challenges requires careful data curation, the development of more sophisticated algorithms that can handle data imbalance, and efforts to ensure that datasets are as complete and representative as possible. Only by overcoming these obstacles can AI‐driven drug repurposing reach its full potential, leading to the discovery of novel therapeutic applications and ultimately improving patient outcomes.

Drug repurposing involves leveraging vast amounts of data from various sources, including genetic, clinical, pharmacological, and biomedical databases. Each of these sources offers unique insights, and integrating them can provide a comprehensive view that is crucial for identifying new therapeutic uses for existing drugs. To enhance the reliability and effectiveness of drug repurposing algorithms, researchers must implement strategies such as data imputation, robust algorithm development, resampling techniques, and the use of appropriate evaluation metrics. These strategies are crucial for accelerating the discovery of new therapeutic uses for existing drugs, ultimately advancing the field of drug repurposing. Therefore, the practical application of AI algorithms in drug repurposing necessitates enhanced data integration to boost the accuracy and applicability of predictive models. By integrating diverse data types, AI models can capture the intricate relationships between drugs, diseases, and patient characteristics, leading to more accurate predictions. Techniques such as data harmonization and normalization are critical in ensuring that data from different sources can be seamlessly combined and analyzed. Furthermore, establishing collaborative platforms with modular functionalities can significantly advance drug repurposing research. Such platforms enable researchers to share data, methods, and findings, fostering a collective approach to solving complex problems. By enhancing data integration and developing advanced machine learning models, researchers can improve the accuracy and applicability of predictive models. Collaborative platforms further support this endeavor by enabling the sharing of resources and knowledge, thereby accelerating the discovery of new therapeutic uses for existing drugs. These strategies, when effectively implemented, can transform the landscape of drug repurposing, offering new hope for personalized medicine and improved patient outcomes.

### 4.2 Limitations of Virtual Screening Algorithm for Drug Repurposing

The efficiency of AI models, once trained, can quickly evaluate new compounds, greatly speeding up the drug discovery process. However, high‐precision deep learning algorithms are often accompanied by high complexity, and compiling large, well‐annotated datasets is expensive and time‐consuming, and high data dependency will result in incomplete or biased data that can lead to inaccurate models. For supervised learning, there is also the risk of overfitting, where the model performs well on training data but poorly on new, unseen data, which also poses a limitation, affects the generalization of predictions, may struggle to discover entirely new drug uses beyond the scope of its training data, and misses innovative reuse opportunities. Unsupervised learning models, on the other hand, do not require labeled data, making them advantageous for dealing with large, complex datasets. These models excel at uncovering hidden patterns and relationships within the data, leading to the discovery of novel drug‐disease associations. Techniques such as clustering and dimensionality reduction can handle diverse data types, revealing insights that might be overlooked by supervised models. This flexibility and capability to identify novel patterns make unsupervised learning a valuable tool in drug repurposing. Despite these strengths, unsupervised learning models face significant challenges, particularly in terms of interpretability and validation. The results from these models can be difficult to interpret, as they do not provide clear explanations for the patterns they identify. Without labeled data, validating the findings and ensuring the accuracy and meaningfulness of the predictions can be challenging. Additionally, unsupervised models generally have less predictive power compared to supervised models, which can limit their effectiveness in specific outcome predictions, such as drug efficacy.

The interpretability of AI‐based predictions remains a significant challenge, particularly in high‐stakes domains like drug repurposing. AI models, especially deep learning algorithms, are often criticized as “black boxes” due to their complex architectures and the lack of transparency in their decision‐making processes. While these models can generate accurate predictions by analyzing vast amounts of data, they rarely provide clear explanations for how specific outcomes are derived, which creates a disconnect between the computational output and the biological or clinical rationale, as clinicians must often justify their treatment decisions based on evidence that aligns with established medical practices. Moreover, the inability to explain predictions poses regulatory challenges. Drug repurposing often requires stringent validation and approval processes, where regulators demand not only evidence of efficacy but also a clear understanding of the underlying mechanisms. If AI models cannot elucidate the biological or pharmacological basis of their predictions, the likelihood of gaining regulatory approval diminishes, delaying or even halting the clinical implementation of promising repurposing candidates. Efforts are being made to address these issues by developing interpretable AI frameworks, such as attention mechanisms, feature attribution techniques, and rule‐based models, which aim to shed light on the factors influencing predictions. By enhancing transparency, these approaches can bridge the gap between computational methods and clinical practice, fostering greater acceptance of AI in healthcare. However, achieving a balance between model complexity, predictive accuracy, and interpretability remains a significant hurdle, highlighting the need for ongoing innovation in this field.

While both supervised and unsupervised learning models have their respective advantages and challenges, their combined use and future advancements promise to significantly enhance the field of drug repurposing. Increased collaboration between pharmaceutical companies, academic institutions, and regulatory bodies will be essential in facilitating data sharing and resource pooling, accelerating the pace of drug repurposing research. For instance, unsupervised learning could be used to identify potential drug candidates, which supervised learning models then validate and predict their efficacy. The development of integrative data platforms that combine genomic, proteomic, clinical, and pharmacological data will provide richer contexts for AI models to analyze, enhancing their predictive capabilities. Advances in AI algorithms, such as deep learning and reinforcement learning, will further improve the discovery and validation of new drugs. The evolution of regulatory frameworks to better accommodate AI‐driven drug discovery and repurposing will ensure that new therapies are safe, effective, and quickly brought to market. Additionally, the development of more interpretable AI models will address the black‐box problem, making it easier for researchers to understand and trust the predictions made by these models. By overcoming data heterogeneity, improving model interpretability, and fostering collaborative research, AI can lead to more efficient discovery and development of novel therapeutic options, refining screening processes by learning from past mistakes, reducing the number of false results, and improving the reliability of predictions. The integration of these advanced computational methods with experimental data will pave the way for a new era in drug repurposing, ultimately benefiting patients with faster access to effective treatments.

### 4.3 The Prospect of Personalized Medicine

In order to enhance treatment efficacy, personalized medicine seeks to match patients with specific repurposed drugs based on individual characteristics, such as genetic profiles or molecular markers.^[^ ^110^ ^]^ As an important part of precision medicine, AI‐driven drug repurposing plays a critical role by facilitating the development of highly personalized treatments. A key opportunity lies in the intersection of AI and personalized medicine, where AI can rapidly and efficiently analyze vast amounts of biomedical data. Machine learning and deep learning models excel at processing complex datasets, including genetic, proteomic, metabolomic, and clinical information. This enables the identification of new therapeutic uses for existing drugs by revealing patterns and relationships that traditional methods might overlook. Furthermore, AI's ability to integrate and analyze data from diverse sources is particularly promising. By holistically considering patient‐specific data—such as electronic health records, genetic profiles, imaging data, and lifestyle information—AI can provide a comprehensive understanding of a patient's health. This approach allows for the discovery of drug repurposing opportunities tailored to the unique characteristics of individual patients, enhancing treatment efficacy and minimizing the risk of adverse effects. By harnessing the power of AI to analyze large‐scale, multidimensional datasets, researchers can identify specific patient subgroups that may benefit from repurposed drugs, thereby refining treatment strategies and improving outcomes. Moreover, AI can help streamline clinical trials by identifying optimal patient populations and predicting responses to treatment, ultimately accelerating the translation of personalized therapies from bench to bedside. As AI continues to evolve, it will increasingly enable a more targeted approach to medicine, bringing us closer to the realization of precision healthcare that is both effective and accessible.

Despite these opportunities, several challenges must be addressed to fully realize the potential of AI in drug repurposing for personalized medicine. Personalized medicine relies on sensitive personal health information, and the integration of diverse data sources increases the risk of data breaches. Obtaining clinical evidence is particularly challenging for oncology and orphan drugs because they are often approved through expedited review pathways and use data from single‐arm trials.^[^ ^111^ ^]^ Consequently, the effectiveness and comparative effectiveness were often uncertain at the time of approval. Moreover, these trials often had relatively short follow‐up periods and evaluated short‐term treatment outcomes. This raises concerns about the clinical benefits of the drugs, especially in the long term.^[^ ^13^ ^]^ Ensuring robust data protection measures and adhering to regulatory requirements such as the General Data Protection Regulation (GDPR) is essential to maintain patient trust and comply with legal standards. Additionally, ethical considerations around the use of AI in healthcare, including informed consent and transparency in AI decision‐making processes, must be carefully managed. Furthermore, the regulatory landscape for AI‐driven drug repurposing is still evolving. Regulatory agencies like the FDA are developing frameworks for the approval and oversight of AI‐based medical products, but the rapid pace of technological advancement presents a challenge to keeping these frameworks up to date. Navigating the regulatory environment requires ongoing collaboration between AI developers, regulatory bodies, and healthcare providers to ensure that AI‐driven solutions are safe, effective, and ethically sound. Current initiatives to screen existing drugs for new uses come mainly from small biotech companies or academic research groups. It takes the combined efforts of public‐private partnerships, nonprofits, academic researchers, and companies to successfully study and approve drugs for other indications.

AI has emerged as a transformative force in the field of drug repurposing, providing unprecedented technical support by efficiently processing large‐scale medical databases, predicting interactions between drugs and novel targets, and optimizing the potential for drug conversion and reuse (**Table** **2**). The development of an AI‐driven, integrated drug repurposing framework could revolutionize the identification of new therapeutic applications for existing drugs, leading to more effective and cost‐efficient treatment options. This approach promises to significantly shorten the traditional drug development cycle, reduce costs, and accelerate the discovery of new therapies. By offering researchers and clinicians a powerful tool, AI enhances their ability to identify potential treatment options more quickly and efficiently than ever before. In addition to technical challenges, the rapid development of AI‐driven drug repurposing raises important ethical questions, particularly regarding the equitable distribution of the benefits of this technology across different populations and regions. There is a risk that the advantages of AI‐driven healthcare innovations could become concentrated in wealthier countries or regions, exacerbating existing disparities in access to healthcare. Addressing these ethical concerns will require coordinated efforts from policymakers, researchers, and industry stakeholders to ensure that the benefits of AI‐driven drug repurposing are distributed fairly. Therefore, while AI holds great promise for advancing the field of drug repurposing, realizing its full potential will require overcoming significant technical and ethical challenges. There is an urgent need to develop explainable AI models that provide clear and understandable rationales, facilitating their integration into clinical workflows. By addressing these issues, we can ensure that AI‐driven drug repurposing contributes to more equitable and effective healthcare solutions worldwide.

**Table 2:** AI‐driven one‐stop drug discovery platform.

Tool	Machine learning module	Validation dataset	Year	Ref.
AIxFuse^[^ ^112^ ^]^	Reinforcement learning, active learning	PLIP program	2024	PMID: 38994407
DeepICL^[^ ^113^ ^]^	Interaction perception model	PDBbind	2024	PMID: 38538598
WISER	Weakly supervised representation learning	CODE‐AE, DepMap portal, TCGA	2024	https://doi.org/10.48550/arXiv.2405.04078
MUSE115^[^ ^114^ ^]^	Expectation‐maximization framework	SHS27K, BioSNAP, DrugBank	2024	PMID: 38796523
ASGARD^[^ ^115^ ^]^	Single‐cell Guided Pipeline	Gene Expression Omnibus	2023	PMID: 36813801
HiSIF‐DTA^[^ ^116^ ^]^	Dual encoder architecture	Davis, KIBA, Human	2023	PMID: 37983161
HyperSynergy^[^ ^117^ ^]^	Few‐shot Hypernetwork Prediction	SYNERGxDB, PubChem, CCLE, ArrayExpress (E‐MTAB‐3610)	2023	PMID: 37027608
DeepCE^[^ ^118^ ^]^	Graph neural network, multihead attention mechanism	LINCS L1000	2021	PMID: 33796820
KGE_NFM^[^ ^119^ ^]^	Combining knowledge graph and via neural factorization machine	Luo's dataset, Hetionet, Yamanishi_08′s dataset and BioKG	2021	PMID: 34811351

## 5 Conclusion

Traditional drug development is a lengthy and expensive process, often taking over a decade and costing billions of dollars. In contrast, repurposing existing drugs that have already been approved for other uses can bypass several stages of the drug development pipeline, since the safety profiles of these drugs are already well‐established. AI‐driven virtual screening can evaluate millions of compounds in a fraction of the time, significantly accelerating the drug discovery timeline by reducing the need for extensive laboratory testing and focusing resources on the most promising candidates, which is crucial in responding to the most promising candidates, and streamlining the drug discovery process. By selecting diverse and representative compounds, AI ensures that the screening process covers a broad chemical space, increasing the likelihood of finding viable drug candidates. For interactions, predictive modes use historical data from known compounds and their interactions to forecast the behavior of new, untested compounds and identify potential side effects and efficacy issues early in the development process. Targeted approaches provide insights into the convergence of drugs on specific molecular entities, while pathway‐based analysis captures the broader systemic effects. Integration of these metrics enriches the understanding of drug mechanisms, offering a multifaceted perspective for identifying candidates with potential for repurposing.

AI in drug repurposing leverages the strengths of both technologies to enhance accuracy, efficiency, and cost‐effectiveness. The time and resources required for training and forecasting computationally intensive network structures may become prohibitive due to the computational intensity of large data sets, and lightweight workflows have gradually become an important consideration for model optimization and improvement. Leveraging these insights can accelerate the discovery and development of new treatments, maximizing the therapeutic potential of existing drugs. The strategic application of mechanistic insights in drug repurposing not only addresses unmet medical needs but also provides a cost‐effective and time‐efficient pathway for drug development.

## Conflict of Interest

The authors declare no conflict of interest.

## Author Contributions

Z.W. conceived and drafted the manuscript. Z.W. and Y.L. drew the figures and summarized the tables. Z.W. and X.S. reworked the paper frame and updated the figures. X.S. and X.H. standardized formatting and representation. Z.W., T.C., and P.Z. discussed the concepts of the manuscript. P.Z. and Y.C. supervised the studies and approved the version to be submitted.
